# Self-Care for Management of Secondary Lymphedema: A Systematic Review

**DOI:** 10.1371/journal.pntd.0004740

**Published:** 2016-06-08

**Authors:** Janet Douglass, Patricia Graves, Susan Gordon

**Affiliations:** 1 College of Public Health, Medical and Veterinary Sciences, Division of Tropical Health and Medicine, James Cook University, Townsville, Queensland, Australia; 2 James Cook University and World Health Organization Collaborating Centre for the Control of Lymphatic Filariasis, Soil Transmitted Helminths and Other Neglected Tropical Diseases, Cairns, Queensland, Australia; 3 College of Public Health, Medical and Veterinary Sciences, Division of Tropical Health and Medicine, James Cook University, Cairns, Queensland, Australia; 4 College of Health Care Sciences, Division of Tropical Health and Medicine, James Cook University, Townsville, Queensland, Australia; Washington University School of Medicine, UNITED STATES

## Abstract

**Background:**

Lymphedema is a debilitating and disfiguring sequela of an overwhelmed lymphatic system. The most common causes of secondary lymphedema are lymphatic filariasis (LF), a vector-borne, parasitic disease endemic in 73 tropical countries, and treatment for cancer in developed countries. Lymphedema is incurable and requires life-long care so identification of effective lymphedema management is imperative to improve quality of life, reduce the burden on family resources and benefit the local community. This review was conducted to evaluate the evidence for effective lymphedema self-care strategies that might be applicable to management of all types of secondary lymphedema.

**Methodology/Principal Findings:**

Searches were conducted in Medline, CINAHL and Scopus databases in March 2015. Included studies reported before and after measures of lymphedema status or frequency of acute infections. The methodological quality was assessed using the appropriate Critical Appraisal Skills Program checklist. Descriptive synthesis and meta-analysis were used to evaluate effectiveness of the outcomes reported. Twenty-eight papers were included; two RCTs were found to have strong methodology, and overall 57% of studies were rated as methodologically weak. Evidence from filariasis-related lymphedema (FR-LE) studies indicated that hygiene-centred self-care reduced the frequency and duration of acute episodes by 54%, and in cancer-related lymphedema (CR-LE) home-based exercise including deep breathing delivered significant volume reductions over standard self-care alone. Intensity of training in self-care practices and frequency of monitoring improved outcomes. Cultural and economic factors and access to health care services influenced the type of intervention delivered and how outcomes were measured.

**Conclusions/Significance:**

There is evidence to support the adoption of remedial exercises in the management of FR-LE and for a greater emphasis on self-treatment practices for people with CR-LE. Empowerment of people with lymphedema to care for themselves with access to supportive professional assistance has the capacity to optimise self-management practices and improve outcomes from limited health resources.

## Author Summary

Secondary lymphedema is a major cause of disability worldwide. The most common causes are treatment for cancer or infection with lymphatic filariasis. In both cases, the lymphatic system is damaged and unable to perform its normal function of removing extracellular fluid and wastes. Protein rich fluid accumulates in the affected area, and if left untreated may progress to ‘elephantiasis’, which is characterised by a grossly enlarged limb and thickening of the skin. Lymphedema is incurable, requires lifelong care, and psychosocial support, but early intervention and good self-management practices can halt progression, preserve quality of life and maintain the ability to participate in normal work and social activities. Current approaches to treatment vary depending on the setting. In developed countries, cancer related lymphoedema is therapist-based and aims to intervene early and prevent disease progression. Lymphatic filariasis related lymphedema is associated with poverty, affecting people living in developing countries where minimal intervention is recommended or available for early stages. By identifying useful practices that can be transferred across cultural and economic borders, people living with lymphedema can be empowered to care for themselves and improve their long term outcomes.

## Introduction

Lymphedema is a high protein edema which forms when the lymphatic system is chronically overwhelmed. Earlier fluid rich stages progress gradually toward enlargement and fibrosis of the subcutaneous compartment and hyperkeratosis of the skin (elephantiasis) [1]. This can occur as a result of congenital factors (primary lymphedema) but is more commonly caused by an alteration in normal lymphatic function leading to secondary lymphedema. The majority of secondary lymphedema occurs through infection with a vector-borne, parasitic disease known as lymphatic filariasis (LF) which is endemic in 73 tropical countries where is it closely associated with poverty [2]. In developed countries lymphedema is more commonly a consequence of some cancer treatments which involve lymph node removal or irradiation. Global estimates of filariasis-related lymphedema (FR-LE) are 16.7 million cases [3] and cancer-related lymphedema (CR-LE) is estimated to affect between 15% and 80% of all cancer survivors [4]. Annual mass drug administration (MDA) of anti-filarial chemotherapy can prevent future transmission of LF [5] and improvements in surgical management of cancer should reduce the incidence of new CR-LE cases [6] but in both aetiologies onset of chronic symptoms may be delayed for months, years or even decades after exposure to the risk [4, 7]. People with any impairment to lymphatic function bear a lifelong risk of developing secondary lymphedema [7].

Lymphatic vessels remove circulating fluid and large molecules from the extracellular spaces of almost all body tissues and transport them to the lymph nodes. This is essential for continuous clearance of pathogenic elements crossing the skin barrier and entering the subcutaneous compartment and in other tissues it is vital in maintaining correct extracellular fluid balance. Cleaned and filtered lymph is returned to systemic circulation via the vascular system. In lymphedema, when normal lymph transport is impeded, protein rich fluid accumulates, mostly in the subcutaneous compartment. Risk of infection is increased; namely acute dermato-lymphangio-adenitis (ADLA) in FR-LE, and cellulitis or erysipelas in CR-LE. Infection then exacerbates disease progression and as lymphedema advances, symptoms become increasingly disabling and disfiguring [8]. In areas endemic for LF, lymphedema causes social stigma, superstition and loss of opportunity to marry [9]. People with CR-LE report depression, poor quality of life and an inability to engage in paid employment [10].

Although essentially the same chronic disease, treatments for FR-LE and CR-LE follow different guidelines. The World Health Organization recommends community based home care (CBHC)[5, 11–13] to improve hygiene and reduce ADLA episodes (the main cause of lost working days) in FR-LE. The program promotes frequent washing and drying of affected areas with particular attention to entry lesions (potential sites of fungal and bacterial infection), passive elevation and range of motion (ROM) exercises, and the use of oral antibiotic or anti-inflammatory medications during acute events. Self-massage and compression bandaging are recommended in advanced stages but usually not implemented in resource-poor settings [14]. In contrast the gold standard for CR-LE is a two phase program with an initial, intensive period of therapist based treatment applying specialized lymphatic massage and multilayer compression bandaging to reduce limb size; followed by an ongoing maintenance phase of self (or partner) lymphatic massage with regular use of compression garments [15]. Meticulous skin care and remedial exercises are a component of both phases.

Established lymphedema is considered to be irreversible, necessitating lifelong care, and family and psychosocial support [1]. Effective management of either FR-LE or CR-LE can improve quality of life for the individual, reduce the burden on family resources and benefit the local community. In developing countries there may be few or no health care services available for people with FR-LE and resources allocated to CR-LE treatment in many developed countries are also insufficient [16, 17]. Poor access to lymphedema health services in both settings and limited evidence of treatment efficacy has resulted in misinformation and unproven management practices. Approaches to management will ultimately be shaped by access to resources and cultural, financial and political influences [17], but effective core strategies that can be applied across cultural and economic borders need to be identified. This review was undertaken to evaluate the outcome of current self-care interventions for FR-LE and CR-LE. Differences and similarities were assessed with respect to self-care components included in the intervention, outcome measures used and the extent of any support services and monitoring. The results have illuminated beneficial practices that may inform health systems in any setting to increase the effectiveness of self-care strategies for people with secondary lymphedema.

## Methods

The Preferred Reporting Items for Systematic Reviews and Meta-Analyses (PRISMA) statement [18] guided the methodology of this review (protocol registration number CRD42013004850). The research question and search terms were determined using the PICO model (population, intervention, comparison, outcomes) [19]. **Participants** were defined as people with established FR-LE or CR-LE. **Interventions** were any type of self-care procedure that could be performed by an individual or within the family group and these included different self-care protocols or specific components of self-care such as self-massage or resistance exercise. **Comparisons** were either between groups in studies with more than one study group performing self-care, or before- and -after cohort studies (controlled or uncontrolled). **Outcomes** were at least one of either a change in objective measure of lymphedema status (such as lymphedema stage, limb volume or limb circumference) or change in frequency or duration of acute episodes (ADLA or cellulitis). Additional optional outcomes were functional or perceived disability, and self-reported symptoms.

### Search strategy

Searches were conducted in Medline (OVID), CINAHL and Scopus databases in October 2013 and updated during March 2015. Medical Subject Headings (MeSH) were used in the Medline search and keywords for the Scopus and CINAHL searches were derived from the Medline MeSH terms to ensure consistency of search terms (Table 1). The search strategy was limited to publications in English and grey literature was not searched. Reference lists of studies and reviews, World Health Organization (WHO) summary reports and editorials were searched to find other original peer reviewed studies. After removal of duplicates the title and abstract of returned studies were screened by two authors (JD, SG). Full inclusion and exclusion criteria used are detailed in S1 Tables.

**Table 1:** Keywords used to search databases.

Search	Keywords
Search 1	((lymphoedema or lymphedema or elephantiasis) and (filariasis))
Search 2	((lymphoedema or lymphedema or elephantiasis) and (cancer or oncology))
Search 3	1 OR 2
Search 4	(self-care or "self care" or basic-care or "basic care" or "community based home care" or "community-based home-care" or "self management" or self-management or self-treatment or "self treatment" or self-massage or "self massage" or "partner massage" or home-care or "home care" or limb-care or "limb care" or foot-care or "foot care" or hygiene or breathing or exercise)
Search 5	3 AND 4

#### Types of studies

The Australian National Health and Medical Research Council (NHMRC) hierarchy of evidence [20] advocates randomised controlled trial (RCT) as the most appropriate study design to answer clinical questions such as the effectiveness of self-care for lymphedema. However, ethical issues may prevent comparison of basic self-care to no self-care and no such trials were found. Therefore, this review included randomized or quasi-randomized comparisons of different types of self-care, as well as before and after cohort studies (controlled or uncontrolled). Studies, including RCTs, where the primary outcome was assessment of a drug or therapist based intervention were accepted if before and after measures for any self-care group(s) was included. When such a study had only one group which met the inclusion criteria for the review, the results of this group were considered during data extraction as for an uncontrolled before and after cohort study.

Studies were excluded if they employed electrical devices, compression pumps or any equipment such as swimming pools which would not be readily available in an average household. Studies were also excluded if the intervention was not solely self-care i.e. required a surgical procedure, was applied by a therapist, or if exercises were performed in instructor led classes. Studies where the primary outcome was quality of life (QOL), socioeconomic factors or evaluation of program implementation were included only if they also reported objective measures of lymphedema status or frequency of infection.

### Assessment of study quality

Two authors (JD, SG) independently appraised the included studies for methodological quality using the Critical Appraisals Skill Program (CASP) RCT appraisal checklist or cohort appraisal checklist appropriate to the study design [21]. A rating of strong, moderate or weak was awarded to each study according to the number of ‘yes’, ‘can’t tell’ and ‘no’ responses. Studies without ‘no’ or with two or less ‘can’t tell’ answers were considered methodologically strong. Studies with one ‘no’ or three ‘can’t tell’ answers were rated moderate and studies with two or more ‘no’ answers rated weak [22]. When the independent appraisal process was completed, studies which did not achieve the same rating by both reviewers were discussed until agreement was reached. A third reviewer (PG) was available where discrepancies could not be resolved but was not required.

### Data extraction and synthesis

One author (JD) independently extracted study data including number of participants, intervention, outcomes, outcome measures, and results. Special characteristics of gender, age, country/setting, lymphedema cause, affected site and stage (or grade) of lymphedema were noted. Data were extracted for change in lymphedema status and effect on acute episodes. Where results were reported consistently with appropriate estimate of precision, a meta-analysis was performed using Review Manager version 5.3 (The Nordic Cochrane Centre, 2014). Data on effect size and outcomes of clinical importance were extracted for narrative synthesis and to highlight differences between the FR-LE and CR-LE studies.

## Results

**Fig 1:**
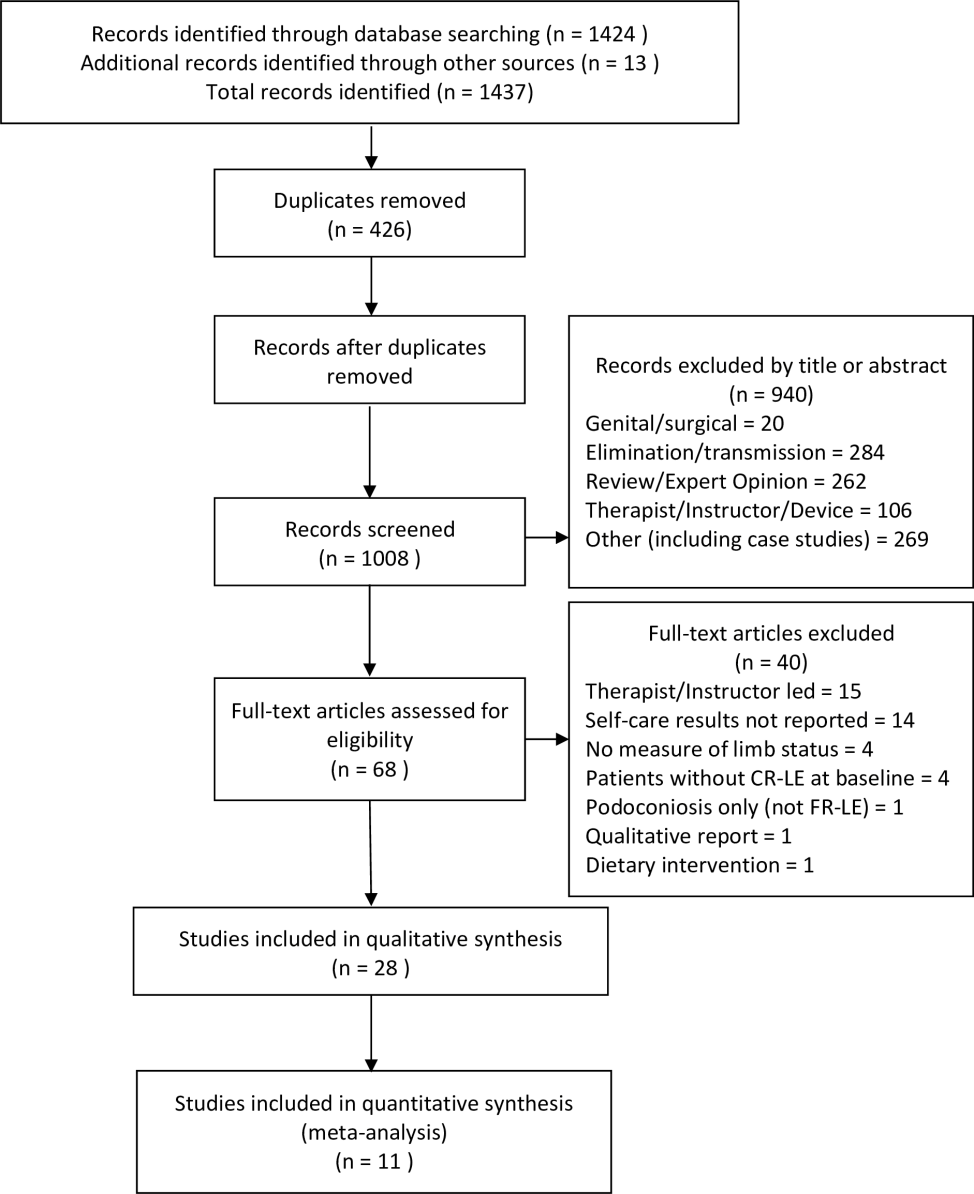
PRISMA flow chart of search results.

Combined searches including reference lists, hand searches and expert referrals yielded 1008 unique articles of which 940 were excluded by title or abstract. Sixty-eight full text articles were screened with 28 meeting the criteria for inclusion. Fig 1 shows the PRISMA flow diagram of study selection and details exclusions by criteria.

### Study design

Ten RCT’s including one quasi RCT and one randomized cross over trial were included. Of these, three FR-LE trials [23–26] and two CR-LE trials [27, 28] compared two or more groups performing different self-care protocols. The remaining five RCTs included only one group using self-care alone and these results were considered as uncontrolled, prospective cohorts [29–33]. Most cohort studies were prospective: ten on FR-LE, including two reports on the same cohort [34, 35], and four on CR-LE. There was one retrospective follow up on a previous FR-LE RCT and two retrospective reports on CR-LE.

#### Quality rating and risk of bias

Two RCT’s were rated as strong but only one of these compared two groups that could be included for review [24]. Of the five RCTs rated as moderate one had two groups performing self-care [27] and four included only one group which met the inclusion criteria. The main risks of bias among the RCTs were inadequate blinding, disparity of participants at baseline and unequal treatment of groups. No cohort studies were rated as methodologically strong and five were rated as moderate quality [35–39]. Risks of bias included: recruitment by convenience sampling, insufficient consideration of confounding factors, low measurement sensitivity and large loss to follow up. Overall 16 studies (57%) were rated as weak methodological quality. The methodological quality rating of each study is provided in S1 Tables.

### Population

There were eighteen reports on seventeen studies about FR-LE and all were conducted in tropical countries endemic for *Brugia malayi* or *Wuchereria bancrofti*. All participants had leg lymphedema, three included people with arm lymphedema [14, 25, 40] and two did not specify the affected limb [33, 41]. Both genders were represented with a greater proportion of females (42.5% - 87%) and although children were admitted in 14 studies most participants were adults with a mean or median age between 35 and 57 years (range 10 to 98 years). Sample sizes ranged between 14 and 1578 with 14 studies of more than 90 participants.

In contrast to FR-LE, most participants in the ten CR-LE studies were women with arm lymphedema after breast cancer and only one RCT included males and participants with leg lymphedema [27]. Other than one Indian cohort [42] all were conducted in developed countries; sample sizes were smaller and four studies had less than 30 participants (range 18–138). Compared to FR-LE studies, the mean or median age of participants in CR-LE studies was higher, between 47 and 66 years (range 25–87 years) and none included adolescents. Population characteristics for all studies are provided in S2 Tables.

### Assessment of limb status

Staging (or grade) of lymphedema was based on clinical assessment of limb size and skin changes. FR-LE studies used either the WHO criteria of Grades 1–3 [43], WHO criteria of Grades I–IV [44] or a seven stage criteria developed by Dreyer, Addiss [11]. CR-LE studies did not state the staging criteria used. Two CR-LE [29, 42] and three FR-LE studies [32, 36, 39] excluded participants with later stages of disease and participants with lymphedema stage 0 were included in two FR-LE studies [30, 36]. The water displacement method [45], which is considered the international gold standard [1], was used to calculate limb volume in both CR-LE and FR-LE studies. Only studies on CR-LE used electronic measuring devices, specifically; multi-frequency bio-impedance spectroscopy which measures extracellular fluid loads [46] and perometry which calculates limb volume using a truncated cone formula from circumferences at 3 millimetre intervals [47]. Limb circumference by tape measure was included in most studies and reported as limb volume, calculated using a truncated cone formula at four or five centimetre intervals, as raw circumference values at fixed points or as a combined average of all points. Studies on unilateral lymphedemas frequently reported on relative limb volume (difference between the affected and unaffected limbs) and percentage change in relative limb volume (RLV) over time. Methods used to assess limb status in each study are provided in S2 Tables.

### Interventions

#### Basic self-care

Basic self-care in FR-LE studies centred on meticulous skin care including frequent washing and drying of the affected limb with soap and water, limb elevation while sleeping and during the day when possible, range of motion (ROM) exercises and application of topical creams to entry lesions. Hygiene equipment such as bowls, soap and towels were provided in nine studies [23, 32, 35, 36, 39, 40, 48–50]. Medicated topical creams and oral antibiotics or anti-inflammatories were recommended or supplied for treatment of ADLA in all prospective studies. In CR-LE studies meticulous attention to skin integrity took the form of regular use of emollients and attention to nail care rather than instruction in washing and drying. Remedial exercises and compression garments were also universally recommended.

#### Additional components of self-care

After basic self-care, the most studied intervention was a home based exercise program on women with CR-LE after breast cancer which included resistance exercise (using gravity or light weights to provide resistance against muscular contraction) [28, 31, 42, 51], pole walking [52], yoga [38] or deep breathing with gentle arm exercise [53]. Self-lymphatic drainage (SLD) is a form of gentle self -massage used to promote the flow of lymph and was taught or recommended in several studies [27, 29, 37, 38], one RCT compared selected essential oils blended into the massage medium to plain cream [27]. Three RCTs on FR-LE compared medicated soap [23] or medicated cream [24, 25] to plain soap or cream in the daily self-care routine and two included one group who used antiseptic ointment daily [30, 33]. Compression therapies were recommended in all CR-LE studies, either as continued use of a previously prescribed garment [27, 31, 38, 42, 53] or a new compression sleeve supplied at study commencement [28, 29, 51, 52, 54]. One FR-LE study trained participants in self-bandaging and provided custom made garments [37] while another recommended the use of compression but reported infrequent or no use among participants [14]. No CR-LE studies utilized self-bandaging.

#### Instruction in self-care

Most self-care protocols were delivered to individuals and their families or carers in outpatient clinics or in-home settings and ranged from a single hour of education and demonstration [29, 32, 38] to daily training over 4 days [37]. Those studies which stipulated a published protocol for morbidity management of FR-LE followed the WHO Community Based Home Care guidelines [14, 34, 35] or the ‘New Hope for People with Lymphoedema’ booklet by Dreyer, Addiss [11] [32]. Six FR-LE and three CR-LE studies received printed instructions [14, 36, 37, 40, 41, 49] [28, 38, 42] and two CR-LE studies delivered home support through digital media [31, 38].

#### Monitoring and follow-up

Participants in most FR-LE studies attended fortnightly or monthly home or clinic visits with extra, surprise field checks in five studies [24, 25, 30, 36, 48]. Some participants were able to access a clinic or health care worker as needed [41, 50] while others recorded ADLA events in a log book [32, 49]. Participants in all prospective CR-LE studies attended measurement clinics at intervals of between two and twelve weeks. Overall FR-LE interventions were of longer duration, typically 12 months (range 4.5–36 months), five followed participants for 12 months after the intervention ceased [24, 25, 32–35] and one was a retrospective follow up on participants in a previous trial by Shenoy, Kumaraswami [25] after 12 months of unsupervised self-care [55]. Compared to FR-LE studies CR-LE studies were generally shorter (range 1–6 months) and only two studies followed subjects for 12 months [29, 54].

### Effects of interventions

#### Basic self-care for FR-LE

*Effect of basic self-care on ADLA in FR-LE*. Eleven studies reported on this outcome (n = 2954). Episodes of ADLA were assessed in a clinic, recorded in a log book or by participant report. Before and after periods were unequal in some studies which reported baseline ADLA using patient recall of the previous 12 months and then collected data as frequently as every two weeks during the intervention.

#### Proportion of persons experiencing ADLA episodes

**Fig 2:**
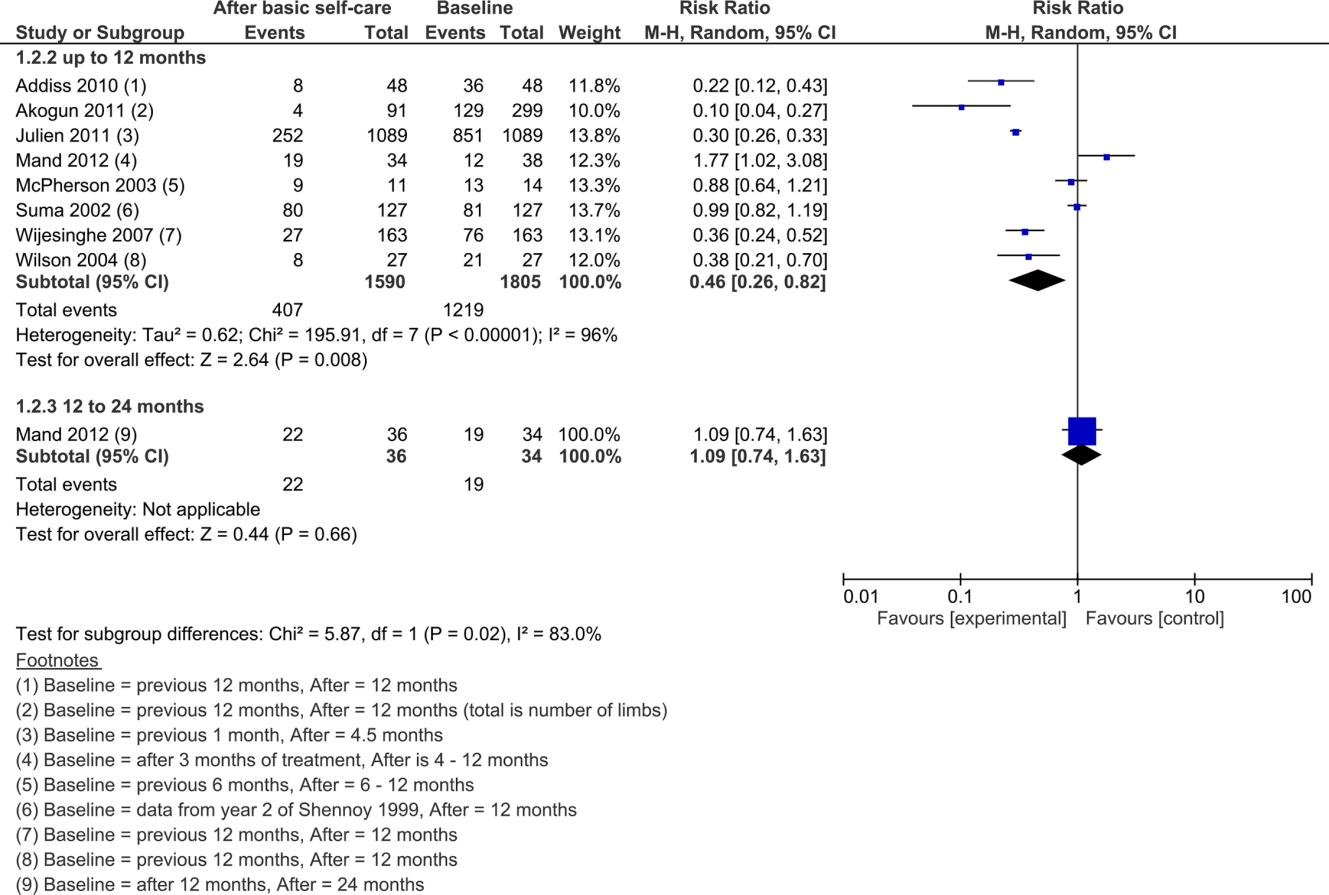
Forest plot of the proportion of FR-LE participants experiencing any ADLA episodes.

Results of eight studies were combined to estimate the effect of basic self-care on ADLA episodes (Fig 2). A random effects model was used due to a high level of heterogeneity between results (I^2^ = 96). Overall, the proportion of participants reporting any ADLA attacks was reduced by 54% (RR = 0.46; 95% CI 0.26 to 0.82). Five studies reported significant reduction in the proportion of participants who experienced any ADLA [14, 36, 39, 40, 50] and two found non-significant reductions [41, 55]. Only the placebo drug group in an RCT reported an increase in persons experiencing any ADLA over 24 months (RR = 1.09, 95% CI 0.74, 1.63) [32]. In the pseudo-RCT which compared three service delivery methods the proportion of participants who reported no episodes in the preceding 6 months increased from 6% at baseline to 93.5% after 12 months [50].

#### Frequency and duration of ADLA

Seven studies reported on this outcome. Six prospective cohorts (n = 1471) found that ADLA frequency or duration reduced between 26% and 100% after 6 to 24 months of self-care [35, 39, 48, 50] and this was significant in two cohorts [14, 36]. Where ADLA incidence was reported by lymphedema stage the best improvements were among participants with stages III and IV (81.3%, p = 0.022) and stage II (80.8%, p<0.001) [14] and more than 60% reduction was achieved in all stages after 6 months [35] and 12 months [48]. Four studies found either; a decrease in the mean duration of ADLA episodes (range 24% - 37.5%) [14, 48] or in the number of working days lost (mean 39%, range 28% - 44%) [34]. In the pseudo RCT the proportion of participants who had experienced ADLA that lasted more than 4 days in the previous year dropped by 78.6% and although 23.4% had reported ADLA that lasted seven days or more at baseline, by the end of the study this had reduced to none [50]. The retrospective follow up of participants in a previous trial by Shenoy, Kumaraswami [25] showed that some benefits were lost 12 months after monitoring ceased and mean ADLA incidence had increased by 65% [55], however this was still 40% lower than before commencement of self-care in the original RCT. The study by Mues, Deming [35] also found that although the significant reductions in ADLA achieved at 6 months (60%) were partially lost after 12 months, the rate remained lower than at baseline (35%) and this was maintained at 24 months. Results of basic self-care on frequency and duration of ADLA episodes are provided in S3 Tables.

### Effect of basic self-care on FR-LE status

Seven studies (n = 1073) reported on change in either objective assessment of lymphedema stage or limb volume, or participant perception of limb status. WHO staging criteria was used more frequently (four studies) than the seven stage Dreyer system (two studies). Limb volume was quantified by water displacement (three studies) or limb circumference (three studies).

#### Changes in limb volume

In a study which excluded participants with stages 5–7 using the Dreyer, Addiss [11] criteria, limb volume reduced in stages 0–4 after 12 months and this was significant in stages two and three which reduced by 4% and 17.1% respectively [36] (Table 2). In a cohort with unilateral leg lymphedema participants who reported a perceived reduction or no change in limb volume (54%) were more likely to have earlier stage FR-LE whereas participants who reported a perceived increase (46%) were more likely to have later stages of disease [48].

**Table 2:** Change in FR-LE limb volume after 12 months of self-care in Addiss et al 2010 [36].

Stage #	Baseline Mean ml (range) *	After 12 months Mean ml (range) *	N (legs)	% reduction	P
**0**	1610 (1080–2160)	1604 (1310–2130)	26	0.3%	
**1**	1937 (1515–2760)	1786 (1470–2030)	9	7.8%	
**2**	1986 (1450–2835)	1909 (1230–2970)	41	4.0%	p<0.05
**3**	2839 (1920–3700)	2354 (1580–3260)	15	17.1%	p<0.05
**4**	3644 (2390–4760)	3082 (1510–4010)	5	15.4%	

# Dreyer et al 2002 [11]

ml = millilitres

* Water displacement method

#### Change in lymphedema stage

Three reports on two studies (n = 533) found a significant proportion of participants had reverted to a lower stage after 12 [14] and 24 months [34, 35] of basic self-care. This effect was greater in participants with early or moderate stage FR-LE, whereas the proportion of people with more advanced disease either stayed the same [35] or increased slightly (not significant) (Table 3). Other studies reported no change in lymphedema stage [32, 39].

**Table 3:** Proportion of Participants by FR-LE stage after 6–24 months of self-care.

Study ID	Stage	Baseline	6 months	12 months	24 months
**Mues et al 2014[35] and Budge et al 2013[34]^1^**	1–2	48.73%	54.01%	55.76%	60.13% p = 0.0064
	3–4	37.57%	32.41%	30.22%	25.32% p = 0.0006
	5–6	12.70%	13.58%	14.02%	14.56%
**Wijesinghe et al 2007[14]^2^**	I	8.6%	n/a	16%*	n/a
	II	52.7%	n/a	46%	n/a
	III	31.3%	n/a	31%	n/a
	IV	7.4%	n/a	8%	n/a

*Eleven people reverted from Stage II to Stage I (p = 0.012)

1 = Stages per Dreyer et al 2002 [11]

2 = Stages per WHO 2003 [13]

### Effect of basic self-care on perceived disability and QOL in FR-LE

Two studies reported on this outcome using either the Dermatology Quality of Life Index (DQLI) [56] or the WHO Disability Assessment Schedule II (WHO DAS II) [57]. Both studies used the seven stage criteria for FR-LE and reported significant improvement in either; all stages after 12 months (n = 14) [41] or in stages 3–7 after 24 months (n = 370) [34]. Results for changes in perceived disability and quality of life are provided in S3 Tables.

### Basic self-care for CR-LE

No study assessed the effect of self-care alone on CR-LE.

### Self-care using topical medication for FR-LE

Five studies (n = 460) used medicated creams or soap in the daily self-care protocol. Three RCTs were used to compare either medicated soap [23] or medicated cream [24, 25] to plain soap or plain cream and medicated ointment was used daily by one self-care group in two other trials [30] [33]. All interventions were of 12 months duration and three studies followed participants for a further year after the intervention ceased [24, 25, 33].

### Effect of self-care with topical medication on frequency and duration of ADLA

Neither of the RCTs which compared medicated cream to plain cream found any between group differences. After the 12-month intervention a significant reduction in annual ADLA episodes of between 63.83% [25] and 77.57% [24] was recorded by all groups and at 24 months the mean annual incidence was still significantly lower than at baseline by 59.52% and 65.02% respectively. During the follow up year, annual incidence in the groups who had used plain cream during the intervention continued to reduce, whereas both groups who had used antibiotic cream experienced an increase (not significant). The trial which compared antibiotic soap to plain soap also found no difference between groups and reported results as for a single cohort [23]. This cohort and the medicated cream groups in two trials (n = 340) all reported significant reductions in mean ADLA episodes of between 62.5% and 65.6% after 12 months [23, 30, 33]. One group was followed for 12 months after the intervention and an overall reduction of 73% from baseline was recorded [33]. Results of the effect of medicated cream or soap on ADLA are shown in S3 Tables.

### Effect of self-care with topical medication on FR-LE status

**Fig 3:**
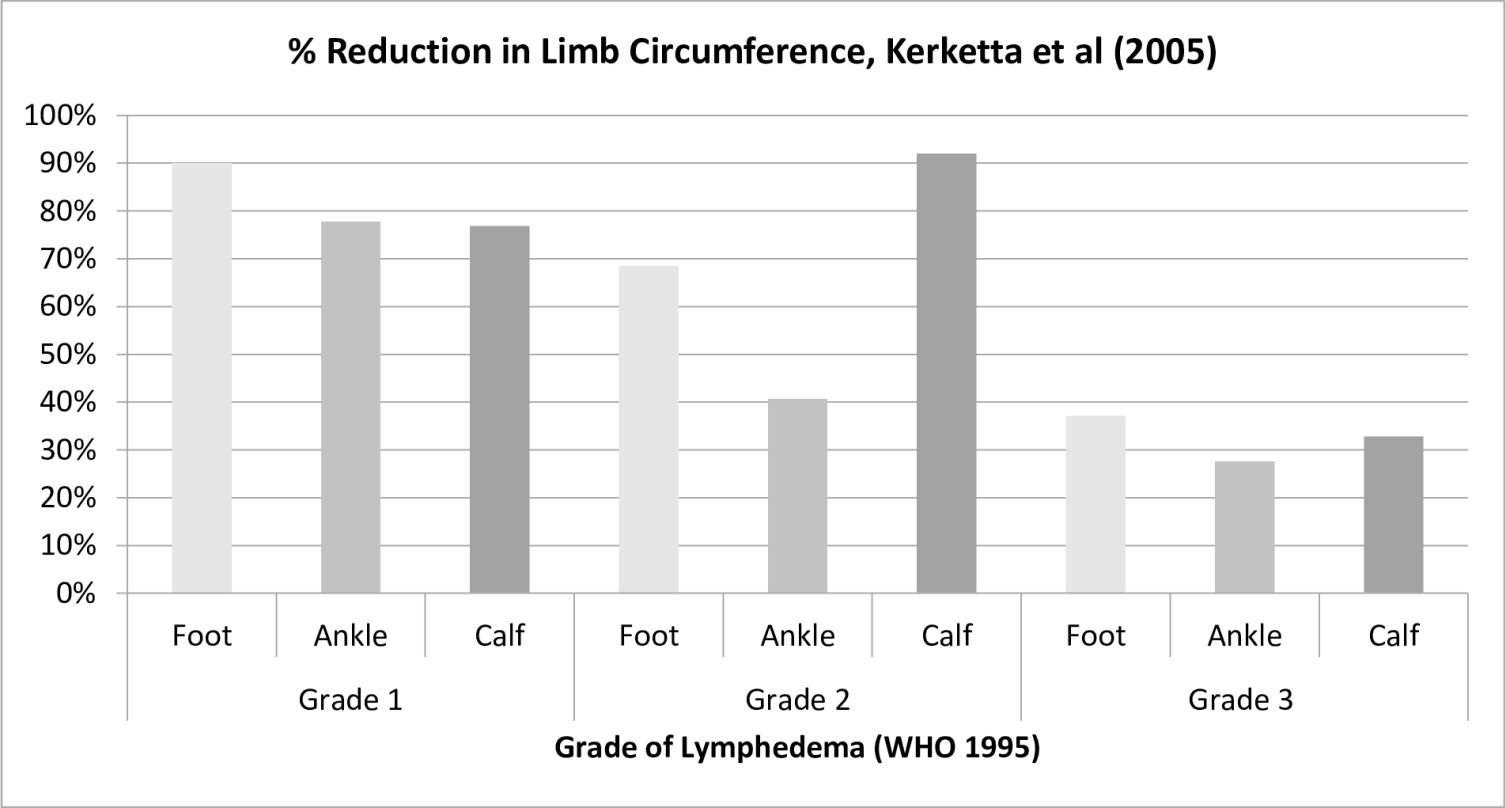
Change in FR-LE limb circumference after 12 months of medicated cream use.

Two trials reported on this outcome (n = 120). In a 12-month intervention on unilateral leg lymphedema [30], raw circumference values for the affected limb reduced between 27.6% - 92% with the greatest reduction at the calf of participants with Grade 2 FR-LE (WHO grades 1–3 [43]) and the least reduction at the ankle in participants with Grade 3 (Fig 3). In this trial the difference in circumference between affected and unaffected limbs also reduced significantly at all time points. In the trial which used water displacement to measure limb volume at baseline and then again during ADLA [24], limb volume increased during 80% of episodes and remained elevated in 73% of cases after two weeks.

### Self-care using topical medication for CR-LE

No CR-LE studies investigated the addition of medicated creams or soaps.

### Home based exercise for FR-LE

No studies assessed the effect of prescribed exercises on FR-LE.

### Home based exercise for CR-LE

Seven studies assessed exercise interventions of between eight weeks and six months duration. All participants were women with unilateral arm lymphedema after breast cancer (n = 197) and six studies reported significant benefits in at least one outcome.

### Effect of home based exercise on CR-LE status

#### Change in limb volume

**Fig 4:**
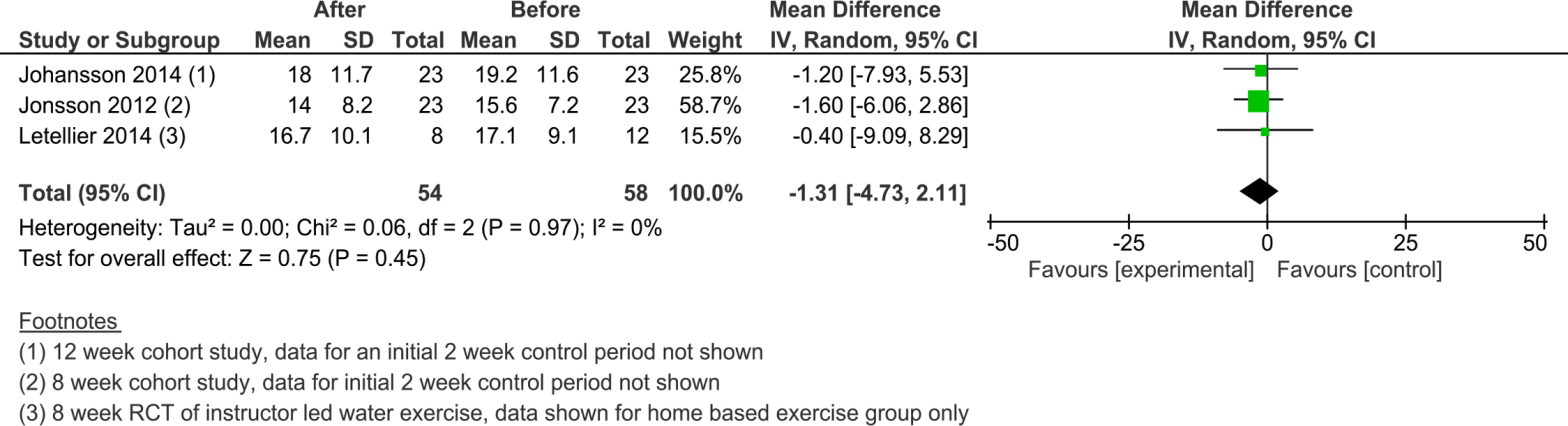
Forest plot of percentage change in relative CR-LE limb volume after exercise.

Results of three studies that reported on change in relative limb volume (RLV) (difference between the affected and unaffected limbs) were combined to estimate the effect of home exercise (Fig 4, n = 54). A random effects model was used due to a high level of heterogeneity between results (I^2^ = 0). Overall, RLV reduced by 1.31% (95% CI -4.73, 2.11).

Statistically significant reduction in limb volume was recorded 10 minutes after commencing a deep breathing exercise with gentle arm movements [53], after eight weeks of pole walking [52] or isotonic arm exercises with deep breathing [42], after ten weeks of weight lifting [51] and in both groups after 12 weeks of either gravity resisted exercise or self-care with hand pumping [28]. In the latter trial both groups had reduced further after six months but this was significant only in the gravity resisted exercise group. This trend was supported by a reduction in arm volume in the home based exercise group in an eight week trial [31] and in a group of women who practiced yoga at home for six months after an initial four week intervention [38] whereas the participants who discontinued yoga practice had experienced an increase (not significant). Results of the effect of home based exercise on limb volume are given in S4 Tables.

### Effect of home based exercise on limb function, self-reported symptoms and QoL

Seven studies (n = 295) reported improvement in one or more of these outcomes.

#### Change in limb function

Significant improvements were found in grip strength [31], muscle strength [51] and cardiovascular fitness [52] but not in ROM [28]. Three studies reported an improvement in participant perception of limb function using various versions of the Disabilities of the Arm, Shoulder and Hand (DASH) questionnaire [58] and this was significant in the home exercise group in one RCT [31].

#### Change in self-reported symptoms

Likert scales and visual analogue scales were used to assess participant perception of symptoms in the affected limb in four studies (n = 143). Significant improvement was found in; pain, heaviness, tightness, pins and needles, and perception of limb size after one month of deep breathing with gentle arm exercise arm [53] and in arm tightness after eight weeks of pole walking [52].

#### Change in quality of life

Two studies on home based exercise reported significant improvement in all domains of the Short Form 36 Health questionnaire after eight weeks [42] and in the Functional Assessment of Cancer Therapy–Breast (FACT-B) after 12 weeks [31].

### Self-lymphatic drainage (SLD) and compression therapies for FR-LE

Only one FR-LE study emphasized the use of SLD and instructed participants in daily self-bandaging. After three weeks of self-bandaging they were fitted with compression garments which were then worn daily for the remainder of the study [37]. Although the self-care protocol in the study by Wijesinghe, Wickremasinghe [14] recommended compression it was not routinely used and results of that study were reported in the basic self-care section.

### Effect of SLD and compression on FR-LE status

#### Change in limb volume

**S**ignificant reductions were recorded after three, six and nine months of performing comprehensive self-treatment including compression therapy (n = 33 legs) [37]. Results for this study are provided in S3 Tables.

### Self-lymphatic drainage (SLD) and compression therapies for CR-LE

One study on both arm and leg lymphedema [27] and two studies on arm lymphedema [29, 54] included SLD in the self-care program (n = 121). The RCT with the mixed arm and leg population investigated the benefits of using Aromatherapy (essential oils) in the massage medium compared to plain cream but found no significant difference between groups and reported most results as a single cohort using SLD [27]. No CR-LE studies included self-bandaging but regular use of a compression garment was recommended in all studies. New garments were supplied at commencement in four studies [28, 29] of which two [51, 52] allowed a control period before the intervention to adjust for the effect of the garment.

### Effect of SLD and compression on CR-LE status

#### Change in limb volume

Two studies that used SLD with compression garments [27, 54] and one group of women that had mild arm lymphedema and used SLD without compression [29] reported significant reduction over three and six months of between 2.59 and 60% (Table 4, n = 121). In the six-month trial of SLD on people with both arm and leg lymphedema, significantly more participants experienced an improvement in limb volume than got worse [27].

**Table 4:** Change in CR-LE limb volume (ml) after 3–6 months of SLD.

Study ID	Limb |Duration (months)|		Before Intervention		After Intervention		% Reduction (95% CI)
	Limb		Volume ml	N	Volume ml	N	
**Anderson et al 2000[29]**	Upper limb (RLV)^2^ Median (range)	3	361 (78–1184)	22	n/a	22	60% (43% - 78%)
**Barclay et al 2006[27]**	Upper limb (RLV)^1^ Median (range)	6	107.0 (-372.0–2421.0)	81	60.0 (-334–2344)	71	43.92%
**Barclay et al 2006[27]**	Right lower limb (WLV)^1^ Mean (SD)	6	6218.3 (1772.5)	81	6057.1 (2093.0)	71	2.59%
**Barclay et al 2006[27]**	Left lower limb (WLV)^1^ Mean (SD)	6	6177.9 (1857.9)	81	5797.9(2014.9)	71	3.20%
**Koul et al 2007[54]**	Upper limb (WLV)^1^ Mean	3	2685	18	2587	18	24% p<0.0001

WLV = Whole limb volume

RLV = Relative limb volume, difference between affected and unaffected limbs

1 = Volume calculated from limb circumference at 4cm intervals

2 = Volume calculated from limb circumference at 5cm intervals

SD = standard deviation

n/a = data not provided

### Effect of SLD and compression on self-reported symptoms and wellness in CR-LE

The Measure Yourself Outcome Profile 2 [59] was used to assess quality of life in participants performing SLD with or without Aromatherapy and both groups reported significant improvements at all time points up to 6 months [27].

## Discussion

In reviewing the evidence for self-care in FR-LE and CR-LE, marked differences were apparent both between and within settings and some key opportunities for improvements were identified. Evidence from the FR-LE population showed that basic self-care alone is effective in preventing ADLA which is consistent with results of a recent review of the effect of hygiene based interventions on FR-LE [60]. Hygiene alone may halt disease progression but is less likely to reduce limb volume and evidence from the CR-LE population indicated that greater volume reductions are achieved when activities such as progressive resistance exercise are included in the self-care routine. Whilst basic self-care was the primary intervention in almost 60% of studies on FR-LE, no CR-LE studies assessed basic self-care alone; rather the self-care group when included were always as controls. Best practice guidelines in CR-LE management are still dependent on therapist performed interventions; however evidence from the FR-LE population suggests that more effort to involve CR-LE patients in their own self-treatment may relieve the financial burden of therapist based care in this population.

The exclusion of any group in comparative studies that received drug or therapist based interventions meant that although ten RCTs were reviewed the bulk of evidence came from observations of a single cohort in studies rated of moderate or weak methodological quality where only one or two groups were performing self-care. There was also inconsistency between assessment techniques and reporting methods. Therefore a review which includes all interventions for FR-LE and CR-LE may provide data for more rigorous meta-analysis. None the less, this first review to systematically examine the similarities and differences in self-care for CR-LE and FR-LE has opened a pathway for further investigation of transferrable strategies for lymphedema management in disparate settings.

The available resources in each study setting were reflected in the simplicity or complexity of devices used to measure change in limb status. All CR-LE studies used water displacement, bio impedance spectroscopy, perometry or a truncated cone formula at small intervals to quantify limb volumes. These methods can detect very small changes which, although statistically significant, might be of minimal clinical significance. In contrast most FR-LE studies relied on less precise measures and less than one quarter used either water displacement or a truncated cone formula. A further 18% of studies used three or four fixed circumference points to compare affected and unaffected limbs or summed or averaged these measures. These methods, especially summed or averaged circumferences lack the precision to detect small changes in limb volume and this could account for variations in reported outcomes between the CR-LE and FR-LE groups. More frequently, FR-LE studies relied on assessment of lymphedema by stage and the use of criteria with only three or four groups was common, since even studies which used the seven stage criteria often grouped them into early, moderate or late stage disease. These graduations may lack the precision to detect small changes and participants who changed from a higher to a lower stage or vice versa may not have always been detected, thereby under or over estimating the effectiveness of the intervention. Most studies tried to minimise inter- or intra-observer variation in staging but the subjective nature of these assessments may also have contributed to the disparate results. These limitations may explain why some studies reported reduction in limb volume without a corresponding change in lymphedema grade. Overall studies which used more precise measuring protocols more often reported significant evidence for volume reduction in both settings.

Less than 12% of FR-LE studies investigated the effect of self-care on subjective symptoms or functional deficits whereas this was reported in 70% of CR-LE studies. The evidence for improvement was weak, mainly due to the disparate range of measuring tools employed, but the overall trend was that a reduction in limb volume was accompanied by improvements in symptoms, perceived disability, overall wellbeing and quality of life. This suggests that self-reported parameters could be used as a proxy for objective measures when these are not available and provide valuable information about the lived experience of FR-LE.

Oral or topical medications for ADLA were used in almost all FR-LE studies but the influence of these could not be adequately separated from the effect of other components of self-care and it is unclear what bearing this had on the results. Although groups treated specifically with oral antibiotic or deworming medications were excluded from the data synthesis some reports showed that the placebo drug groups performing basic self-care had better long term results than groups that had initially received oral medications [24, 25, 33]. Studies on topical medications for ADLA showed no additional benefit over self-care using placebo creams or soaps and the necessity of prophylactic oral medication for ADLA remains controversial. Similarly, in CR-LE studies the use of a compression sleeve was considered an integral aspect of self-care but few studies controlled for the effect of a new garment or included frequency of garment use in the statistical analysis. Where this was done the effect of the compression sleeve was shown to be significantly correlated with improvement in all groups. Whilst these treatments might be considered to be core elements of self-care, access to medication in poorly resourced settings and non-adherence to compression therapies in CR-LE warrant the investigation of self-care protocols that do not rely on these components.

Effective self-care implementation requires some degree of education, instruction or demonstration and the role of the educated health worker or trained volunteer cannot be ignored. FR-LE studies which provided frequent monitoring and support were associated with greater improvements than studies which offered minimal or no support services. The study by Suma, Shenoy [55] which retrospectively reviewed participants in a previous drug based RCT indicated that without monitoring, program effectiveness is lost over time, an effect also found in a later follow up of the study by Addiss, Louis-Charles [36] which could not be included in this review [61]. Ultimately, the long term success of any self-care intervention will depend on individual ownership of and adherence to the daily self-care practices and the level of family or local support available. This was demonstrated clearly in the study by Akogun and Badaki [50] where one group was able to alter the program design to suit their immediate cultural and social constraints and reported good outcomes, whereas the two groups who could not alter the program design to suit their personal circumstances experienced a large loss to follow up.

Despite the wide variation in measurement techniques and support services, it was apparent that hygiene-centred, basic self-care can reduce the frequency and duration of ADLA episodes by approximately 50%, and this was particularly beneficial for people with later stages of disease. There was less evidence for a reduction in limb volume but the study by Joseph, Mony [24] showed that ADLA led to an increased limb volume which persisted after the infection had been treated and Mues, Deming [35] demonstrated that ADLA is related to days of work lost. Thus a reduction in ADLA episodes without change in limb size may still improve outcomes for many individuals. This was illustrated histologically in the study by Wilson, Guarner [39] which showed that basic self-care improved skin integrity and prevented new infections while limb stage remained the same. Where a reduction in limb volume was reported in FR-LE, greater benefits were experienced among participants with early stages, suggesting that implementation of a self-care routine as soon as lymphedema is detected has the potential to curtail the number of cases that progress to advanced stages. Current guidelines for FR-LE will not assist program managers to find and address these earliest stages of lymphedema yet this may be the optimal time to intervene in terms of volume reduction and long term prevention of ADLA. Basic hygiene is enough to control ADLA but this review has shown that limb volume is more difficult to reduce in later stages regardless of the setting [27, 48]. Advanced lymphedema is characterised by fibrosis and fatty induration of the tissues which become much more difficult to reduce than in early stages where the swelling is more characteristically due to protein rich fluid. So to reduce limb volume more intervention is required at an earlier stage than is currently indicated in the WHO guidelines.

Reduction in limb volume was reported in all CR-LE studies, all of which included at least one additional component of self-care. Since publication of the 2005 study by Moseley, Piller [53], specific deep breathing exercises appear frequently in interventions for CR-LE as was evidenced by their inclusion in six studies in this review. Home based exercise, including deep breathing, is easy to perform, require no financial resources, can be continued alone after minimal initial instruction and may contribute to overall improvement in health and wellbeing, but exercise advice included in the current protocols for FR-LE is very limited. Simple resistance exercise and deep breathing could be easily incorporated into CBHC particularly in cultures where activities such as Yoga or Tai Chi may be readily available and acceptable and the addition of such components to current WHO recommendations warrants investigation.

In FR-LE, issues pertaining to infection, wet environments and lack of foot wear make compression therapies problematic. However, Bernhard, Bernhard [37] and other studies that could not be included in this review [62] have showed that people with FR-LE are capable of performing complex compression bandaging and daily use of compression garments. This requires a greater initial investment in the training and education of people with FR-LE so that they have a better understanding of the purpose and effect of each treatment component. The long term benefit of this increased investment was demonstrated in the study by Bernhard, Bernhard [37] where limb volume reduction was significantly greater in the self-treating group compared to the therapist treated group (results for the therapist treated group were not included in this review). This supports consideration of compression therapies in FR-LE when possible and although the evidence is limited, also suggests that self-bandaging could be further explored for people with CR-LE.

Although people with arm lymphedema were included in several FR-LE studies, results were not reported by limb. Only the CR-LE study by Barclay, Vestey [27] reported results by affected limb and in this study the impact of SLD on volume reduction was much greater for arms than legs. This limited comparison of results by limb implies that interventions may deliver different results depending on the location of the lymphedema. Similarly, some studies allowed participation of children as young as five years old but no study gave an analysis by age to determine if children had better or worse outcomes than the adult subjects, nor was the effect of gender explored. It is possible that age and gender influence self-treatment outcomes and investigation of different components of self-care by limb, age and gender should be considered.

Since disability from existing LF will continue to increase for several decades even after transmission has been successfully interrupted, and increasing cancer survivorship is a primary focus of cancer research, it is probable that the incidence of new lymphedema cases from both causes will continue to increase for the foreseeable future. Focussing efforts toward greater emphasis on early intervention and prevention has the potential to alleviate this future burden. Implementation of morbidity management in the global effort to eliminate LF requires evidence based strategies to attract and maintain funding, and reducing the burden of CR-LE for all cancer survivors requires more research about lymphedema of the leg. In both cases high quality studies that investigate reversal of early stage disease and analysis of individual components of self-care by age, gender, stage and location of lymphedema are essential to determining optimal, financially sustainable, management.

## Supporting Information

S1 Tables Inclusion criteria and methodological quality of assessed papers. (DOCX)

S2 Tables Population characteristics of all studies. (DOCX)

S3 Tables Effect of interventions on Filariasis Related Lymphedema (FR-LE). (DOCX)

S4 Tables Effect of interventions on Cancer Related Lymphedema (CR-LE). (DOCX)
