# Supplementary material for: Self-Care for Management of Secondary Lymphedema: A Systematic Review
Source: PLoS Negl Trop Dis. 2016 Jun 8;10(6):e0004740. doi: 10.1371/journal.pntd.0004740 (PMC4898789; doi:10.1371/journal.pntd.0004740)
Supplement: S1 Tables — (DOCX) [file pntd.0004740.s001.docx]

**S1 Tables: Inclusion Criteria and Methodological Quality of Assessed Papers**

Table S1.1: Inclusion and Exclusion criteria as registered on the PROSPERO database

| **Category** | **Inclusion Criteria** | **Exclusion Criteria** |
| --- | --- | --- |
| **Participants** | Participants living in developing countries with lymphedema secondary to infection with lymphatic filariasis.  Participants living in developed countries with lymphedema secondary to treatment for cancer. | Participants who have genital only lymphedema without involvement of at least one limb.  Participants who have previously received surgical interventions as part of lymphedema management. |
| **Intervention** | Any intervention or combination of interventions given for the treatment of lymphedema that can be performed by the participant or a family member.  Any intervention of combination of interventions given to address factors contributing to progression of lymphedema that can be performed by the participant or a family member. | Mass drug administration studies in filariasis endemic areas where lymphedema outcomes are not specifically measured.  Studies using benzo-pyrones or other drug treatments which are no longer in use.  Surgical interventions for hydrocele or other surgical interventions for lymphedema.  Studies involving treatment dependent on lymphedema specialist therapist services or instructor led programs.  Studies involving treatment use of specialist lymphedema treatment devices such as compression pumps or custom made garments.  Drug trials where data for a self-care only (placebo) group are not given.  Interventions which address genital lymphedema only and do not provide management for at least one limb. |
| **Types of studies** | Any peer reviewed publication assessing a self-care intervention for secondary lymphedema where pre and post test data evaluating physical changes of the limb has been collected and reported. | Reviews/opinion/editorial articles.  Single case studies (except as part of a cohort study).  Economic evaluations or qualitative studies that do not include pre and post test measurement of lymphedema status. |

Table S1.2: Methodological Quality of papers assessed using the CASP RCT Appraisal Form

| **Author (Year)** | Study Rating | Did the trial address a clearly focussed issue | Was the assignment of patients to treatment randomized | Were all of the participant who entered the trial accounted for at its conclusion | Were patients, health workers and study personnel blind to the treatment | Were the groups similar at the start of the trial | Aside from the experimental intervention were the groups treated equally | Can the results be applied to the local population | Were all clinically important outcomes considered | Are the benefits worth the harms and costs | **Yes Score / 9 questions** |
| --- | --- | --- | --- | --- | --- | --- | --- | --- | --- | --- | --- |
| **Addiss et al 2011** | **W** | Y | Y | N | CT | Y | CT | Y | N | Y | **5** |
| **Akogun & Badaki 2011** | **W** | Y | N | N | N | CT | N | Y | Y | Y | **4** |
| **Andersen et al 2000** | **M** | Y | Y | Y | CT | N | Y | Y | Y | Y | **7** |
| **Barclay et al 2006** | **M** | Y | Y | Y | N | CT | Y | Y | CT | Y | **6** |
| **Joseph et al 2004** | **S** | Y | Y | Y | Y | Y | Y | Y | Y | Y | **9** |
| **Jeffs et al 2013** | **W** | Y | Y | Y | N | Y | Y | N | Y | Y | **7** |
| **Kerketta et al 2005** | **S** | Y | Y | Y | CT | CT | Y | Y | Y | Y | **7** |
| **Letellier et al 2014** | **M** | Y | Y | Y | CT | N | Y | Y | Y | Y | **7** |
| **Mand et al 2012** | **M** | Y | Y | Y | CT | Y | N | Y | Y | Y | **7** |
| **Shennoy et al 1998** | **M** | Y | Y | Y | CT | CT | CT | Y | N | Y | **5** |
| **Shennoy et al 1999** | **W** | Y | Y | Y | CT | N | Y | Y | N | Y | **6** |

S=Strong, M=Moderate, W=Weak

Y=Yes, N=No, CT=Can’t Tell

Table S1.3: Methodological Quality of papers assessed using the CASP Cohort Appraisal Form

| **Author (Year)** | Study Rating | Did the study address a clearly focussed issue | Did the authors use the appropriate method to answer the question | Was the cohort recruited in an acceptable way | Was the exposure adequately measured to minimize bias | Was the outcome accurately measured to minimize bias | Have the authors identified all important confounding factors | Have they taken account for the confounding factors in the design or analysis | Was the follow of subjects complete enough | Was the follow up of subjects long enough | Do you believe the results | Can the results be applied to the local population | Do the results of this study fit with other available evidence | **Yes Score / 12 questions** |
| --- | --- | --- | --- | --- | --- | --- | --- | --- | --- | --- | --- | --- | --- | --- |
| **Addiss 2010** | **M** | Y | Y | N | Y | CT | Y | Y | CT | Y | CT | Y | Y | **8** |
| **Bernhard 2003** | **M** | Y | Y | CT | Y | Y | N | CT | Y | Y | Y | CT | Y | **8** |
| **Budge 2013** | **W** | Y | Y | Y | N | Y | CT | Y | N | Y | Y | Y | CT | **8** |
| **Das 2013** | **W** | Y | Y | CT | Y | N | N | N | CT | Y | CT | Y | Y | **6** |
| **Douglass 2012** | **M** | Y | Y | Y | CT | Y | CT | N | CT | Y | Y | Y | Y | **8** |
| **Gautam 2011** | **W** | Y | N | CT | Y | Y | N | CT | CT | CT | Y | Y | Y | **6** |
| **Johansson 2014** | **W** | Y | Y | Y | Y | N | CT | N | Y | Y | CT | Y | Y | **8** |
| **Jonsson 2014** | **W** | Y | Y | Y | Y | CT | CT | N | N | Y | Y | Y | Y | **8** |
| **Jullien 2011** | **W** | Y | Y | Y | N | CT | N | Y | N | Y | Y | Y | Y | **8** |
| **Koul 2007** | **W** | Y | Y | Y | CT | N | N | CT | N | Y | Y | Y | Y | **7** |
| **Mathieu 2013** | **W** | Y | CT | CT | N | Y | N | N | CT | Y | CT | N | N | **3** |
| **McPherson 2003** | **W** | Y | Y | N | CT | CT | CT | CT | N | Y | Y | Y | Y | **7** |
| **Moseley 2005** | **W** | Y | N | CT | Y | N | N | Y | Y | Y | Y | Y | Y | **8** |
| **Mues 2015** | **M** | Y | Y | Y | N | Y | Y | Y | Y | Y | Y | Y | Y | **11** |
| **Suma 2002** | **W** | Y | Y | Y | Y | CT | N | N | Y | Y | Y | Y | Y | **9** |
| **Wijesinghe 2007** | **W** | Y | Y | Y | N | CT | Y | N | N | Y | CT | Y | Y | **7** |
| **Wilson 2004** | **M** | Y | Y | CT | CT | Y | CT | Y | N | Y | Y | Y | Y | **8** |

S=Strong, M=Moderate, W=Weak

Y=Yes, N=No, CT=Can’t Tell
