# Supplementary material for: Self-Care for Management of Secondary Lymphedema: A Systematic Review
Source: PLoS Negl Trop Dis. 2016 Jun 8;10(6):e0004740. doi: 10.1371/journal.pntd.0004740 (PMC4898789; doi:10.1371/journal.pntd.0004740)
Supplement: S2 Tables — (DOCX) [file pntd.0004740.s002.docx]

# **S2 Tables: Population Characteristics of All Studies**

Table S2.1: Description of Randomized Controlled trials (RCT) on FR-LE

| **Study** | | **Population** | | **Intervention** | | **Outcomes** |
| --- | --- | --- | --- | --- | --- | --- |
| **Author (Year)**  **Country**  **Setting** | **Design**  **Duration and Follow Up**  **Groups in Review** | **Affected limb (s)**  **Inclusion criteria**  **# significant baseline difference** | **N (% retention)**  **Age**  **Gender** | **Self-Care Program**  **Equipment supplied (if any)**  **Comparator groups** | **Monitoring**  **Measurement Interval** | **Outcome  (Outcome measure)**  ***significant benefits reported** |
| **Mand et al (2012)**  Ghana  21 communities | RCT, 3 groups  Follow up : 24 months  Group 3, n = 54 | Leg (s)  Stages 1-5  Minimum body weight 40kg  Aged 18-60 years | n = 162 (73.5%)  Mean 47.7 years (SD 10.8)  Female = 71.4% | Foot hygiene:  Trained using “New Hope for People with Lymphedema” (Dreyer et al 2002)  Soap, towels and bowls provided | Home visit : 3, 12 and 24 months  Measured : Baseline, 3, 12 and 24 months | Lymphedema Stage (Examination, Dreyer et al 2002)  Limb circumference  (Combined average of 4 points)  ADLA frequency  (Journal record)  Skin thickness  (Ultrasound)  Endothelial Growth Factors (ELISA) |
|  |  |  |  | Daily medication for 6 weeks : Group 1 = Amoxicillin Group 2 = Doxycycline Group 3 = Placebo |  |  |
| **Addiss et al (2011)**  Haiti  Hospital Clinic | RCT, 2 groups  Intervention: 12 months  Group 1, n= 100 Group 2, n = 100 | Leg (s)  Competent in self-care  Reside within 10km radius of the clinic | n = 200 (98.5%)  Mean 37 years (Range 11-77)  Female = 87% | Hygiene and skin care:  Wash legs daily, apply antifungal and antiseptic creams as needed  Soap and creams provided | Home visits: Monthly  Measured at: Baseline and 12 months | ADLA frequency* (Recall previous 12 months)  Compliance to self-care and use of topical creams (Self-report)  Outcomes not reported by group |
|  |  |  |  | Group 1 = Antibacterial soap Group 2 = Plain soap |  |  |
| **Akogun & Badaki (2011)**  Nigeria  3 local government areas | Pseudo RCT,  3 groups  Intervention:  12 months  Group 1, n = 131 Group 2, n = 91 Group 3, n = 103 | Leg (s)  Previous ADLA  # group size, gender, age | n = 325 (30.2%)  ≤19 years = 0.6% 20 - 49 years = 60% ≥50 years = 39.4%  Female = 67.1% | Basic hygiene: Education in limb care and exercise, apply antifungal and antiseptic creams as needed  Equipment and creams provided | Monthly impact monitoring  Measured; Baseline, 6 and 12 months | ADLA frequency and duration (Recall previous 12 months)  Limb status* (Warts, size, folds, odours, lesions) |
|  |  |  |  | Group 1 = Community service delivery model, able to alter delivery design Group 2 = Group leader service delivery model Group 3 = Health centre service delivery model | |  |
|  |  |  |  |  |  |  |
| **Kerketta et al (2005)**  India  8 villages | RCT, 3 groups  Intervention : 12 months  Group 3, n=100 | Unilateral leg  ≥ 1 ALDA previous 12 months  Participants with ADLA treated and then excluded | n = 300 (84.7%)  14 - 65 years  Gender not given | Foot-care: Demonstration, regular limb cleaning, keep dry, clip nails, apply ointment between toes & sides of feet, attend to injuries  Tablets and cream provided | Fortnightly clinic visits Surprise field checks  Measured between 4pm – 8pm at : Baseline, 90, 180 and 360 days | Limb circumference* (3 points)  ADLA frequency*  (Recall previous 12 months) (Recall previous fortnight) |
|  |  |  |  | Medication, twice daily for 12 days every three months : Group 1 = DEC Group 2 = Penicillin Group 3 = Antiseptic ointment (betadine) | |  |
| **Joseph et al (2004)**  India  22 villages | RCT, 5 groups  Intervention : 12 months  Follow up : 12 months  Group 1, n = 30 Group 2, n = 30 | Leg (s) Arm (s)  ≥2 ADLA previous 12 months  ≥ 15 years old  > 30 kg  Stratified by Grade (WHO 1992) | n = 150 (90%)  Mean years (SD) Group 1, 49.8 (12.5) Group 2, 49.9 (11.1)  Female = 75% | Basic hygiene : Intensive training, clipping nails, nightly cleansing, keep dry, 6% salicylic-acid ointment between toes and sides of feet  Tablets and cream provided | Home visits every 3- 4 days  Attend clinic during ADLA  Measured at : Baseline, 12 and 24 months | Limb Volume (Water displacement)  ADLA frequency*, severity and duration (Observed in clinic)  Blood Serology during ADLA (Micro titration)  Skin lesion microbiology (Cultured from swabs) |
|  |  |  |  | Daily medication : Group 1 = Placebo + plain ointment (zinc oxide) Group 2 = Placebo + antibiotic cream (1.5% framycetin)  Group 3 = DEC + plain ointment (zinc oxide) Group 4 = Penicillin + plain ointment (zinc oxide) Group 5 = DEC + Penicillin + plain ointment (zinc oxide) | |  |
| **Shenoy et al (1999)**  India  Hospital Clinic | RCT, 5 groups  Intervention : 12 months  Follow up : 12 months  Group 4, n = 30 Group 5, n = 30 | Leg (s) Arm (s)  ≥2 ADLA previous 12 months | n=150 (95%)  Mean 43 years  (Range 18 - 67)  Female = 54% | Washing Program : Nightly washing, keep dry, clip nails, apply ointment between toes and sides of feet, apply cream during infection or injury  Tablets and cream provided | Attend clinic : Fortnightly or during ADLA  Surprise field visits  Measured at : Baseline, 12 and 24 months | ADLA by grade of lymphedema* (Examination, WHO 1992)  ADLA frequency, duration* (Recall previous 12 months) (Assessed fortnightly) |
|  |  |  |  | Daily medication : Group 1 = Penicillin + plain cream (zinc oxide) Group 2 = DEC + plain cream (zinc oxide) Group 3 = DEC + Penicillin + plain cream (zinc oxide) Group 4 = Placebo + antibiotic ointment (framycetin)  Group 5 = Placebo + plain cream (zinc oxide) | |  |
| **Shenoy et al (1998)**  India  Hospital clinic and surrounding area | RCT, 3 groups  Intervention:  12 months  Follow up:  12 months  Group 3, n = 40 | Limb not specified  Enrolled during ADLA, admitted and treated with oral antibiotics before baseline  ≥2 ADLA previous 12 months  Grade 0-III (WHO 1992) | n = 120 (94%)  Median 40 years (Range 18 – 65)  Female = 42.5% | Local limb care: Wash and dry twice daily, apply antibiotic and antifungal ointments, oral antibiotics and paracetamol during episodes  Tablets and creams provided | Report to clinic: Fortnightly or during ADLA  Measured at: Baseline, 12 and 24 months | ADLA frequency*  (Recall previous 12 months) (Assessed fortnightly) |
|  |  |  |  | Monthly medication, single dose: Group 1 = Ivermectin Group 2 = DEC Group 3 = Placebo |  |  |

#= Statistically significant differences between groups at baseline

*= Statistically significant improvement reported for this outcome

ADLA = Acute dermato lymphangio adenitis

DEC = Diethylcarbamazine

WHO = World Health Organization

ELISA = Enzyme-linked Immunoabsorbent Assay
SD = standard deviation

Table S2.2: Description of Cohort Studies on FR-LE

| **Study** | | **Population** | | **Intervention** | | **Outcomes** |
| --- | --- | --- | --- | --- | --- | --- |
| **Author (Year)**  **Country**  **Setting** | **Design**  **Duration and Follow Up**  **Groups in Review** | **Affected limb (s)**  **Inclusion criteria**  **# significant baseline difference** | **N (% retention) Age**  **Gender** | **Self-Care Program and equipment supplied (if any)**  **Comparator groups (if any)** | **Monitoring**  **Measurement Interval** | **Outcome**  **(Outcome measure)**  ***significant benefits reported** |
| **Mues et al (2015)** India  30 villages* | Prospective  Uncontrolled  Intervention: 24 months | Leg (s)  ≥ 3 months swelling  ≥ 14 years old | n = 370 (85.1%)  Mean 57 years (SD 13.94)  Female = 58.92% | Basic lymphedema management:  Daily washing with soap, daily exercise and elevation and wear footwear outside the home. Topical and oral treatment for infection  Soap and antifungal cream supplied for 6 months | NGO program delivering community based home care (CBHC)  Measured at: Baseline, 1, 2, 3, 6, 12, 18 and 24 months | Lymphedema Stage* (Grouped 1-3, 4-7 Dreyer 2002)  ADLA frequency and duration (Recall previous 30 days) |
|  |  |  |  |  |  |  |
| **Budge et al (2013)**  India  30 villages* same cohort as Budge et al 2013 | Prospective  Uncontrolled  Intervention: 24 months | Leg (s)  ≥ 3 months swelling  ≥ 14 years old | n = 370 (85.1%)  Mean 57.15 years  Female = 58.92% | Home Based Care: Regular limb washing, appropriate exercise, elevation, treat bacterial and fungal infections, use footwear | NGO program delivering community based home care (CBHC)  Measured at: Baseline, 1, 2, 3, 6, 12 and 24 months | Lymphedema Stage* (Grouped 1-2, 3, 4-7 Dreyer 2002)  ADLA  (Recall previous 30 days)  Perceived Disability* (WHODASII)  Lost days of work* (Recall previous 30 days) |
|  |  |  |  |  |  |  |
| **Das et al (2013)**  India  Research Centre | Prospective  Within subject control  Intervention: 12 months | Unilateral Leg  Stratified by Grade  (WHO 1992)  15 – 60 years | n = 97 (87%)  Mean years (SD) I = 37.6 (11.2)  II = 47.3 (11.1)  III&IV = 52.3 (11.8)  Gender not reported | Domiciliary limb hygiene: Trained in twice daily washing and drying, identify entry lesions, apply antifungal creams, oral medication for ADLA, use footwear  Hygiene kit supplied monthly  Affected vs unaffected limb volume | Surprise checks  Measured at: Baseline, 3, 6 and 12 months | Limb volume (Water displacement)  ADLA Frequency and duration (Recall previous 12 months (Observed)  Locomotor function (Indian Government Guidelines) |
| **Mathieu et al (2013)**  Togo  7 endemic areas | Prospective  Uncontrolled  Intervention: 3 years | Leg (s)  Patients with log books available for analysis | n = 341 (55.13%)  Mean 48 years (Range 12 - 98)  Female = 61% | Footcare: Trained in regular washing and drying, elevation, exercise  Soap, towels and illustrated booklet provided | Log book  Home visits:  Twice in the first week, weekly then monthly  Log book reviewed every 3 months | ADLA frequency  (Log book)  Program adherence* (Log book) |
|  | Representative sample of the National program | | |  |  |  |
| **Jullien et al, (2011)**  Africa  People enrolled in the National 'Washing' Project’ | Prospective  Uncontrolled  Mean follow up 4.5 months (range 1-5) | Limb not specified  ≤ 1 clinic visits in 4.5 months  # age of retained vs lost subjects | n = 1578 (69%)  Mean years 46.6 (Range 10 - 98)  Female = 74.50% | Washing Program: Wash and dry limb up to 4 times per day, clip nails, use footwear  Basin, cup, soap & medication and leaflet to identify ADLA provided | Train 1 health care worker per 15 patients  Attend clinic 1-5 times  Measured at: Baseline and clinic visits (range 2 – 6 total visits) | ADLA frequency* (Recall previous month)  Frequency of consultations  (Clinic records)  ADLA by frequency of consultation* |
| **Addiss et al. (2010)**  Haiti  Hospital clinic | Prospective, 2 groups  Intervention: Minimum 6 months  Follow up:  Mean 22.1 months (range 6.3 – 41.2)  Group 2 n = 48 | Leg (s)  ≥ 5 clinic visits  Stage 0-4 (Dreyer 2002)  # Mean ADLA | n = 175 (100%)  Mean 37.3 years (Range 10 – 85)  Female = 82.9% | Hygiene program: Education, wash legs, ROM exercises, elevation, oral and topical medication as required  Equipment, antibiotic cream, instruction booklet provided | Visit clinic: Every 4-6 weeks or during ADLA  Ad hoc home visits  Measured at: Baseline and every clinic visit | ADLA frequency* (Recall previous 12 months) (Observed)  Limb volume* (Water displacement)  Compliance (Questionnaire) |
|  |  |  |  | Group 1 = Therapist treatment  Group 2 = Self-care |  |  |
| **Wijesinghe et al (2007)**  Sri Lanka  Two filariasis clinics | Prospective  Uncontrolled  Intervention: 12 months | Leg(s)  Arm(s)  ≥ 6 weeks swelling  ≥ 5 years old | n = 413 (39.5%)  Arm(s) = 1.7% Leg(s) = 98.3%  ≤ 35 years = 6% 36 - 65 years = 80% > 65 years = 14%  Female = 81.6% | Community Based Home Care: Individual training of patient or carer, twice daily washing, keep dry, elevation, ankle exercises, use footwear, prevent trauma, treat entry lesions, oral and topical medication for ADLA  Patient booklet provided | Visit clinic: Monthly if experiencing symptoms  Measured at: Baseline and 12 months | Change in lymphedema grade*  (Examination, WHO 1992)  Number of entry lesions* (Examination)  ADLA frequency*, duration, and ADLA management practices* (Recall previous 12 months)  Perceived improvement (Interview) |
| **Wilson et al (2004)**  Haiti  Hospital clinic | Prospective  Within subject control  Intervention: Mean days 365 | Leg(s)  No ADLA previous 2 weeks  Stage 1 – 3 (WHO 1992)  Residing within 10km of the clinic | n = 91 (29.7%)  Median 39 years (Range 16 - 75)  Female = 80% | Lymphedema Self-care; Daily washing, ROM exercises, elevation, prevent entry lesions  Antimicrobial medication and symptomatic treatment during ADLA  Soap, towels, wash basin and cream provided | Attend clinic or home visit:  Every 4 – 6 weeks or during ADLA  Biopsy follow up between 317 - 656 days | Lymphedema stage  (Examination, WHO 1992)  ADLA frequency* (Recall previous 12 months) (Observed)  Histologic Changes* (Skin-punch biopsy, 4 mm) |
|  |  |  | Affected vs unaffected histological analysis n = 26 | |  |  |
|  |  |  |  |  |  |  |
| **Bernhard et al (2003)**  Tanzania  Lymphedema clinic | Prospective 2 groups  Group 2, n=25  Intervention: 9 months | Leg(s)  ≥ 6 months swelling  ≥ 18 years old  Able to reach own toes for bandaging | n = 46 (79.7%)  Mean years (SD) Female = 47(14.3) Male = 42(16.6)  Female = 63% | Self-treatment: Instruction, daily wash and dry, SLD, elevate day and night, exercise, use footwear, avoid injury, medical treatment for ADLA  Bandages, compression garments and illustrated leaflet provided | Attend clinic: Weeks 2 and 3 3, 6 and 9 months  Measured at: Baseline, 3 weeks, 3, 6 and 12 months | Limb Volume*  (circumference measures at 4cm intervals)  Entry wounds and fungal infections (Observed) |
|  |  |  |  | Compression bandaging for 3 weeks then ongoing compression garment: Group 1 = Therapist treated Group 2 = Self-treated |  |  |
| **McPherson T (2003)**  Guyana  One municipality | Prospective  Uncontrolled  Intervention:  12 months | Limb(s) not stated  FR-LE patients within the community receiving no care | n=14, FU = 79%  Mean 47 years (Range 21 - 65)  Female = 81.8% | Train a specialist nurse within a hospital clinic  Morbidity reduction: Hygiene, skin-care, elevation, simple exercises, oral antibiotics for ADLA, antibacterial and antifungal cream as needed  Patient education leaflet provided | Nurse available for advice and support  Measured at: Baseline and 12 months | Quality of Life* (Dermatology Quality of Life Index)  ADLA (Recall 6 months)  Stage  (Dreyer et al 2002) |
| **Suma et al (2002)**  India  Hospital Clinic | Retrospective  Uncontrolled  Intervention: 12 months | Leg (s) Arm (s)  Participants of a previous RCT (Shenoy et al 1999) | n = 127  Median 45 years (Range 18 - 67)  Female = 56.7% | Foot-care:  Nightly cleaning, keep dry, clip nails, apply ointment between toes and sides of feet, antibiotic cream as needed, use footwear | Nil monitoring  Baseline data from previous RCT  Measured at 12 months | ADLA frequency (Recall 12 months)  Entry lesions (Clinical assessment) |

#= Statistically significant differences between groups at baseline

*= Statistically significant improvement reported for this outcome

SD = standard deviation
NGO = Non Government Organization
WHO = World Health Organization

WHODASII = WHO Disability Assessment Scale II

ADLA = Acute dermato lymphangio adenitis

ROM = range of motion

ADLA = Acute dermato lymphangio adenitis

Table S2.3: Description of Randomized Controlled Trails (RCT) on CR-LE

| **Study** | | **Population** | | **Intervention** | | **Outcomes** |
| --- | --- | --- | --- | --- | --- | --- |
| **Author (Year)**  **Country**  **Setting** | **Design**  **Duration and Follow Up**  **Groups in Review** | **Affected limb (s)**  **Inclusion criteria**  **^#^ significant baseline difference** | **N (% retention)**  **Age**  **Gender** | **Self-Care Program**  **Equipment supplied (if any)**  **Comparator groups (if any)** | **Monitoring**  **Measurement Interval** | **Outcome**  **(Outcome measure)**  ***significant benefits reported** |
| Letellier (2014)  Canada  University health centre and private lymphedema clinic | RCT, 2 groups  Intervention: 12 weeks  Group 1, n =12 | Unilateral Arm  Stratified by relative limb volume of < 25% / ≥ 25%  # Onset after surgery, duration of lymphedema | n = 25 (72%)  Mean years (SD) Group 1, 53.4 (9.35) Group 2, 56.4 (9.76)  Female = 100% | Home exercise; SLD, corrective and strengthening exercises, compression sleeve  DVD of home exercises provided | Log book  Measured at: Baseline and 12 weeks | Relative limb volume (Water Displacement) (Circumference at 4cm intervals)  Grip strength*  (Dynamometer)  Perceived function* (DASH)  Pain (Short-form Magill questionnaire)  Quality of Life* (FACT –B) |
|  |  |  |  | Group 1 = Home exercise Group 2 = Home exercise and weekly aquatic exercises classes | |  |
| **Jeffs and Wiseman (2013)**  UK  Hospital clinic | RCT, 2 groups  Intervention: 6 months  Group 1, n = 11 Group 2, n = 12 | Unilateral Arm  Relative limb volume ≥ 10% | n =23 (100%)  Median years  (LQ, UQ) Group 1, 66 (51, 63) Group 2, 64.5  (56, 73.5)  Female = 100% | Standard care: Daily including skin-care, exercise, hand pumping, compression sleeve  Home based exercise:  Resistance exercise, deep breathing, compression  New compression garment and illustrated instruction sheet provided | Measured at: Baseline, 4, 12 and 26 weeks | Relative limb volume* (Perometry)  ROM (Goniometer)  Quality of Life (LYMQoL)  Perception of function  (Quick DASH-9)  Adherence (Self-report) |
|  |  |  |  | Group 1= Standard care + Home based exercise Group 2 = Standard care | |  |
|  |  |  |  |  | |  |
|  |  |  |  |  | |  |
| **Barclay et al (2006)**  UK  Hospital clinic | RCT, 2 groups  Intervention: 6 months  Group 1, n = 40  Group 2, n = 41 | Leg(s) Arm(s)  Stratified by age and affected limb  ≥ 18 years  Able to perform self-massage | n = 81 (92.6%)  unilateral arm = 61 bilateral leg = 20  25 - 80 years  Female = 95% | Standard care: Daily SLD, exercise, skin care, compression garment | Measured at: Baseline, 1, 2, 3 and 6 months | Limb volume*  (Circumference at 4cm intervals)  Symptoms and wellbeing* (MYMOP2) |
|  |  |  |  | Daily self-massage: Group 1= Plain massage cream Group 2 = Aromatherapy massage cream | |  |
| **Andersen et al (2000)**  Denmark  Hospital clinic | Randomized crossover trial 2 groups  Intervention: 3 months  Follow up: 12 months  Group 2, n = 22 | Unilateral arm  Relative limb volume ≥ 200ml  # Endochrine therapy | n = 42 (92.86%)  Median 53 years (Range 25 - 77)  Female = 100% | Standard therapy: instruction, SLD, exercise, skin care and safety precautions  Custom made sleeve and glove provided | Measured at: Baseline, 1, 3, 6, 9 and 12 months  Results at 6, 9 and 12 months not included in review | Relative Limb Volume* (Circumference at 5cm intervals)  Self-reported Symptoms (Interview)  Compliance (interview) |
|  |  |  |  | Group 1 = Therapist massage Group 2 = Self-massage |  |  |

#= Statistically significant differences between groups at baseline

*= Statistically significant improvement reported for this outcome

SD = standard deviation
LQ = Lower quartile
UQ = Upper quartile
LYMQol = Quality of Life Measure for Limb Lymphoedema (Keeley et al 2010)

DASH = Disabilities of the arm, shoulder and hand, [www.dash.iwh.on.ca](http://www.dash.iwh.on.ca)
Quick DASH 9 = Disabilities of the arm, shoulder and hand, [www.dash.iwh.on.ca](http://www.dash.iwh.on.ca)
SLD = Self lymphatic drainage (massage)
FACT B = Functional assessment of cancer therapy – breast [www.facit.org](http://www.facit.org)
MMYOP2 = Make yourself medical outcome profile version 2 [www.measuringimpact.org](http://www.measuringimpact.org)

Table S2.4: Description of Cohort Studies on CR-LE

| **Study** | | **Population** | | **Intervention** | | **Outcomes** |
| --- | --- | --- | --- | --- | --- | --- |
| **Author (Year)**  **Country**  **Setting** | **Design**  **Duration and Follow Up**  **Groups in Review** | **Affected limb (s)**  **Inclusion criteria**  **^#^ significant baseline difference** | **N (% retention)**  **Age**  **Gender** | **Self-Care Program**  **Equipment supplied (if any)**  **Comparator groups (if any)** | **Monitoring**  **Measurement Interval** | **Outcome**  **(Outcome measure)**  ***significant benefits reported** |
| **Johansson et al (2014)**  Sweden  Hospital clinic | Prospective  Uncontrolled  New sleeve:  2 weeks  Control period: 2 weeks  Intervention:  12 weeks | Arm unilateral  ≥ 6 months swelling  Relative limb volume ≥ 200ml or  ≥ 2cm  > 70 years | n = 26 (88.5%)  Mean 58 years (SD 8.0)  Female = 100% | Progressive resistance exercise for 4 weeks  Home weight lifting exercise for 8 weeks  New compression garment provided | Fortnightly review of log book  Measured at 2, 4, 6 and 14 weeks | Relative limb volume* (Water Displacement)  Extracellular fluid ratio (Bio impedance spectroscopy)  Isometric Muscle Strength* (Strain gauge)  Grip strength (Hand dynamometer)  Perceived disability (DASH) |
| **Jonsson and Johansson (2014)**  Sweden  Hospital clinic | Prospective  Uncontrolled  New sleeve: 2 weeks  Intervention:  8 weeks | Arm unilateral  >75 years  Relative limb volume ≥ 10%  Palpable tissue changes or subjective symptoms | n = 35 (65.7%)  Mean 60.4 years (SD 8.3)  Female =100% | Pole walking with light arm exercise 3-5 times per week  New compression garment | Log book Fortnightly clinic visit  Measured at baseline, 2, 4, 6, 8 and 10 weeks | Relative limb volume* (Water Displacement)  Cardiovascular fitness* (Bicycle ergonometer)  Perceive disability (DASH)  Self-reported symptoms* (Likert scale)  General wellbeing (Questionnaire) |
| **Douglass et al. (2012)**  Australia  Hospital clinic | Retrospective  2 groups  Follow up: 6 months  Group 1, n = 9  Group 2, n = 9 | Unilateral arm  Participants of a previous RCT on a 4 week yoga program | n = 18  Mean years (SD) Group 1, 65.0 (12.4) Group 2, 60.4 (11.1)  Female = 100% | Self-management: Education, SLD, exercise, skin care, compression sleeve | Baseline (data from RCT)  Measured at 6 months | Relative limb volume (Bio impedance spectroscopy) (Perometry)  Tissue compressibility (Tonometry)  Self-reported symptoms (Likert scale)  Quality of Life (Visual Analogue Scale) |
|  |  |  |  | Home yoga program; Daily exercise, breathing exercise, meditation  Illustrated instruction sheet and CD-Rom of guided relaxation provided  Group 1 = Continued home yoga Group 2 = Discontinued home yoga | |  |
| **Gautam et al (2011)**  India  Hospital clinic | Prospective  Uncontrolled  Intervention: 8 weeks | Unilateral Arm  Relative limb volume ≥ 200ml or  ≥ 2cm  Stage I and II (Criteria not given) | n = 38 (84.2%)  Mean 46.6 years (SD 6.98)  Female = 100% | Self-care;  Education, SLD, skin-care, elevation, avoid injury, compression sleeve | Weekly phone call Clinic or home visit at 4 weeks  Measured at: Baseline, 4 and 8 weeks | Circumference* (Difference between affected and unaffected arm at 4 places)  Relative limb volume* (Water Displacement)  Quality of Life* (Short Form Health Survey 36) |
|  |  |  |  | Home based exercise program: Progressive resistance exercise and deep breathing  Instruction sheet provided | |  |
| **Koul et al (2007)**  Canada  Lymphedema clinic | Retrospective 3 groups  Group 3, n= 18 | Unilateral arm  ≥ 1 year follow up  # severity of lymphedema | n = 138  Mean years 54.3 (range 29 - 82)  Female = 100% | Training in SLD, skin-care and remedial exercise  Custom made compression sleeve provided | Telephone interview at 3 months  Measured at: 6 and 12 months | Relative Limb Volume* (Circumference at 4cm intervals) |
|  |  |  |  | Treatment allocation at discretion of therapists: Group 1 = Therapist treatment including bandaging, deep breathing Group 2 = Therapist treatment without bandaging  Group 3 = Self-care with SLD and deep breathing | |  |
| **Moseley et al (2005)**  Australia  Hospital clinic | Prospective Retrospective 2 groups  Group 1, n= 40  Group 2, n = 28 (Control cases from a previous study) | Unilateral Arm  ≥ 6 months swelling  Relative limb volume ≥ 200ml | n = 68 (76.5%)  Mean years  (SD / range)  Group 1, 60.1  (1.6 / 42.-76) Group 2, 65  (2.0 / 42-87)  Female = 100% | Deep breathing exercise:  Gentle arm extension with each breath, 5 breaths and 1 minute rest, repeated for 5 cycles | Log book  Measured at: Baseline, every 10 minutes first hour, 24 hours, 1 week, 1 month | Relative limb volume* (Perometry)  Truncal fluid (Bio impedance spectroscopy)  Tissue compressibility (Tonometry)  Self-reported symptoms* (Likert scale) |
|  |  |  |  | Group 1 = Deep breathing exercise Group 2 = Retrospective controls |  |  |

#= Statistically significant differences between groups at baseline

*= Statistically significant improvement reported for this outcome

SD = standard deviation

DASH = Disabilities of the arm, shoulder and hand, [www.dash.iwh.on.ca](http://www.dash.iwh.on.ca)

SLD = Self lymphatic drainage (massage)
