# Supplementary material for: Self-Care for Management of Secondary Lymphedema: A Systematic Review
Source: PLoS Negl Trop Dis. 2016 Jun 8;10(6):e0004740. doi: 10.1371/journal.pntd.0004740 (PMC4898789; doi:10.1371/journal.pntd.0004740)
Supplement: S3 Tables — (DOCX) [file pntd.0004740.s003.docx]

# S3 Effect of Interventions on Filariasis Related Lymphedema (FR-LE)

## Effect of Basic Self-Care on ADLA

Table S3.1: Frequency of ADLA episodes after 6 - 24 months of basic self-care

| **Study ID** | **Group(s)** | **Baseline** | | **Treatment Period** | | **% change** |
| --- | --- | --- | --- | --- | --- | --- |
|  |  | **Mean** | **N** | **Mean (range)** | **N** |  |
| **Mues et al 2014** | Self-care cohort | 0.35 * | 370 | 6 months  0.14 | 324 | Reduced 60% |
|  |  |  |  | 12 months  0.23 | 321 | Reduced 35% from Baseline |
|  |  |  |  | 24 months 0.23 | 316 | Reduced 35% from Baseline |
| **Addiss et al 2010** | Self-care cohort | 1.31 (range, 0-6) | 48 | 12 months  0.22 | 48 | Reduced 83% p=0.0001 |
| **Akogun & Badaki 2011** | Community care group | 3.8 | 299 (limbs) | 0 | 91 (limbs) | Reduced 100% |
|  | Patient care group | 2.6 | 137 (limbs) | 0 | 7 (limbs) | Reduced 100% |
|  | Health facility group | 3.4 | 237 (limbs) | 2.5 | 2 (limbs) | Reduced 26% |
| **Wilson et al 2004** | Participants who consented to a second skin biopsy | 1.7 (range, 0-8) | 91 | 0.5 (range, 0-3) | 26 | Reduced 71% |
| **Suma et al 2002#** | Unsupervised year | 1.7 (range, 1-12) | 127 | 2.8 (range, 1-42) | 127 | Increased 65% |
|  | Baseline from 1999 drug trial | 4.7 (SE 0.7) | 150 | 2.8 (range, 1-42) | 127 | Reduced 40% |

** Rate per person month, # Baseline data is from 2nd year of follow up in Shenoy et al 1999, SE = standard error*

Table S3.2: Reduction in frequency of ADLA episodes by Stage of FR-LE after 6 - 24 months of basic self-care.

| **Study** | **LE Stage** | **Baseline** | **N** | **6 months** | **N** | **% Change** | **12**  **months** | **N** | **% Change** | **24 months** | **N** | **% Change** |
| --- | --- | --- | --- | --- | --- | --- | --- | --- | --- | --- | --- | --- |
| **Mues et al 2014*** Rate per person month | 1 – 3 | 0.29 | 317 | 0.1 | 324 | - 65.5% | 0.19 | 321 | - 34.5% | 0.2 | 316 | - 31.0% |
|  | 4 - 6 | 0.78 | 53 | 0.22 |  | - 71.8% | 0.42 |  | - 46.2% | 0.46 |  | - 41.0% |
| **Das et al 2013** Mean episodes | I | 2.4 | 10 |  |  |  | 0.8 | 10 | - 66.7% |  |  |  |
|  | II | 3.4 | 50 |  |  |  | 1.2 | 47 | - 64.7% |  |  |  |
|  | III & IV | 4.8 | 36 |  |  |  | 1.8 | 36 | - 62.5% |  |  |  |
| **Wijesinghe et al 2007**  Mean (SD) episodes | I | 0.36 (0.74) | 14 |  |  |  | 0.21 (0.43) | 26 | - 41.7% |  |  |  |
|  | II | 0.99 (1.68) | 86 |  |  |  | 0.19 (0.54) | 75 | - 80.8% ^1^ |  |  |  |
|  | III & IV | 2.78 (7.92) | 63 |  |  |  | 0.52 (1.56) | 62 | - 81.3% ^2^ |  |  |  |
| **Suma et al 2002^#^** Mean episodes | I | 0.4 | 127 |  |  |  | 1.4 | 127 | + 40.0% |  |  |  |
|  | II | 0.8 |  |  |  |  | 1.6 |  | + 100% |  |  |  |
|  | III | 1.6 |  |  |  |  | 2.8 |  | + 75.0% |  |  |  |
|  | IV | 3.0 |  |  |  |  | 4.4 |  | + 46.7% |  |  |  |

*^1^ p<0.001*

^2^ p=0.022

*# Baseline data from 2n`d year of observation Shenoy et al (1999)
SD = standard deviation*

Table S3.3: Duration of ADLA episodes after 12 months of self-care for FR-LE

| **Study ID** | **Previous 12 months** | | **Treatment Period** | | **% Reduction** |
| --- | --- | --- | --- | --- | --- |
|  | **Mean days** | **N** | **Mean days** | **N** |  |
| **Das et al 2013** | 4 | 93 | 2.5 | 93 | 37.5% |
| **Wijesinghe et al 2007** | 5.8 (SD 3.97, range 1-30, mode 7) | 163 | 5.7 (SD 3.4, range 2-14, mode 3) | 163 | 24.0% RR 0.14 (95%CI 0.94, 0.56) |

*SD = standard deviation*

Table S3.4: Duration of ADLA episodes in the previous 6 months by percentage of participants

| **Study ID** | **Duration in days** | **Baseline** | | **After 12 months** | | **% Change** |
| --- | --- | --- | --- | --- | --- | --- |
|  |  | **% participants** | **N** | **% participants** | **N** |  |
| **Akogun & Badaki 2011** | 0 | 6% | 299 (limbs) | 83.5% | 91 (limbs) | Increased 92.8% |
|  | 1-3 | 39.8% |  | 9.9% |  | Reduced 75% |
|  | 4-6 | 30.8% |  | 6.6% |  | Reduced 78.6% |
|  | ≥7 | 23.4% |  | 0% |  | Reduced 100% |

Table S3.5 Days of work lost in the preceding 30 days after enrolment in a CBHC program

| **Study** | **Stage** | **Time point** | | | **% Reduction** | |
| --- | --- | --- | --- | --- | --- | --- |
|  |  | **Baseline** | **6 months** | **24 months** |  |  |
| **Budge et al 2013** | All stages | 6.4 (95% CI: 5.6, 7.2) | 2.9 (95% CI: 2.4, 3.4) | 3.9 (95% CI: 3.2, 4.6)* | 39% | *sig lower than baseline |
|  | Advanced (4-7) | 10.4 |  | 5.9 | 44% | p=0.0083 |
|  | Moderate (3) | 6.2 |  | 4.5 | 28% | p=0.0439 |

## Effect of basic self-care on Perceived Disability and Quality of Life in FR-LE

Table S3.5: Change in perceived disability and quality of life after 12 – 24 months

| **Study ID** | **Stage*** | **Before** | **N** | **After** | **N** | **Duration** | **Improvement** |
| --- | --- | --- | --- | --- | --- | --- | --- |
| **McPherson T 2003^1^**  **Mean score (range)** | All stages | 10.2 (2-18) | 14 | 4.1 (0-11) | 11 | 12 months | 6.8 (0-15) p≤0.0001 |
| **Budge et al 2013^2^**  **Composite score** | 1 – 2 | 60.1 | 184 | 57.4 | 188 | 24 months | 4.5% p=0.0697 |
|  | 3 | 69.0 | 133 | 62.0 | 82 | 24 months | 10.1% p=0.0011 |
|  | 4 – 7 | 80.2 | 53 | 69.9 | 46 | 24 months | 12.8% p=0.0044 |

** Stages per Dreyer et al 2002*

*1=Dermatology Quality of Life Index (DQLI)*

*2=WHO Disability Assessment Schedule II (WHO DAS II) (WHO, 2001)*

## Effect of self-care with medicated/plain cream or soap for FR-LE

Table S3.6: Reduction in mean ADLA - RCTs on cream or soap

| **Study ID** | **Group** | **Previous year** | | **Treatment year** | | | **Follow up year** | | |
| --- | --- | --- | --- | --- | --- | --- | --- | --- | --- |
|  |  | **Mean** | **N** | **Mean** | **N** | **% Reduction*** | **Mean** | **N** | **% Reduction*** |
| **Joseph et al 2004** | Medicated cream | 2.43 | 30 | 0.56 | 27 | 76.95% ^#^ | 0.85 | 27 | 65.02% ^#^ |
|  | Plain cream | 2.63 | 30 | 0.59 | 27 | 77.57% ^#^ | 0.44 | 27 | 83.27% ^#^ |
| **Shenoy et al 1999** | Medicated cream | 4.2 | 30 | 1.1 | 29 | 73.81% p<0.001 | 1.7 | 29 | 59.52% p<0.001 |
|  | Plain cream | 4.7 | 30 | 1.7 | 28 | 63.83% p<0.001 | 1.0 | 28 | 78.72% p<0.001 |
| **Shennoy et al 1998** | Medicated cream | 3.63 | 40 | 1.25 | 39 | 65.6% p<0.001 | 0.98 | 30 | 73.0% p<0.001 |
| **Kerketta et al 2005** | Medicated cream | 3.2 | 100 | 1.2 | 84 | 62.5% ^#^ | n/a | n/a | n/a |
| **Addiss et al 2011** | Medicated & plain soap | 1.1 | 200 | 0.4 | 197 | 63.3% ^#^ | n/a | n/a | n/a |

** Reduction from baseline
# significance not given
n/a = no follow up in this study*

## Effect of Self-care with SLD and compression on FR-LE

Table 15. Reduction in leg volume (litres)*

| **Study ID** | **Self-bandaging period** | | | **Compression garment period** | | | | | | | |
| --- | --- | --- | --- | --- | --- | --- | --- | --- | --- | --- | --- |
|  | **After 3 weeks** | | **After 3 months** | | | | **After 6 months** | | **9 months** | | |
|  | **Litres (range)** | **N** (legs) | | **Litres (range)** | **N** (legs) | **Litres (range)** | | **N** (legs) | | **Litres (range)** | **N** (legs) |
| **Bernhard et al 2003** | 0.85  (-0.12 to 5.75)  p=0.049 | 33 | | 0.61  (0.12 to 7.55) p=0.003 | 31 | 0.48  (-0.06 to 7.55) p=0.001 | | 32 | | 0.32  (-0.03 to 5.77) p=0.016 | 25 |

** Limb volume calculated from circumference measures at 4 cm intervals*
