# Supplementary material for: Self-Care for Management of Secondary Lymphedema: A Systematic Review
Source: PLoS Negl Trop Dis. 2016 Jun 8;10(6):e0004740. doi: 10.1371/journal.pntd.0004740 (PMC4898789; doi:10.1371/journal.pntd.0004740)
Supplement: S4 Tables — (DOCX) [file pntd.0004740.s004.docx]

# S4 Tables: Effect of Self-Care on CR-LE

**Table S4.1: Effect of home-based exercise on arm volume**

| **Study ID** | **Exercise** | **Duration** | **Intervention Group** | | **Controls** | | **Measure** |
| --- | --- | --- | --- | --- | --- | --- | --- |
|  |  |  | **Change** | **N** | **Change** | **N** |  |
| **Gautum et al 2011** | Isotonic exercise, deep breathing | 8 weeks | Reduced mean 122.83mls  (+/- 30.37) p < 0.0001 | 38 | Within subject control^1^ | n/a | Water displacement |
| **Jeffs & Wiseman 2013** | Gravity resisted exercise | 12 weeks | Reduced 8.08%  p = 0.05 | 11 | Reduced 2.83%  p = 0.041 | 12 | Perometry |
|  |  | 26 weeks | Reduced 11.69%  (95% CI -26.57, -5.12)  p = 0.013 | 11 | Reduced 9.2%  (95% CI -17.71, 1.1) | 12 |  |
| **Moseley et al 2005** | Deep breathing, gentle arm exercise | 10 minutes | Reduced mean 52mls (5.8%) p = 0.004 | 24 | Within subject control^1^ |  | Bio-impedance spectroscopy,  Perometry |
|  |  | 1 month | Reduced 101mls (9%) | 24 | Increased 7ml # | 28 |  |
| **Douglass et al 2012** | Home yoga program | 6 months | Reduced 14.3% | 9 | Increased 25.4% | 9 | Bio-impedance spectroscopy |
|  |  | 6 months | Reduced 9.8% | 9 | Increased 16.7% | 9 | Perometry |

*# Controls from a previous study
1 = Uncontrolled cohort study*
